# Prevalence of major cardiovascular risk factors and adverse risk profiles among three ethnic groups in the Xinjiang Uygur Autonomous Region, China

**DOI:** 10.1186/1476-511X-12-185

**Published:** 2013-12-17

**Authors:** Jing Tao, Yi-tong Ma, Yang Xiang, Xiang Xie, Yi-ning Yang, Xiao-mei Li, Zhen-Yan Fu, Xiang Ma, Fen Liu, Bang-dang Chen, Zi-xiang Yu, You Chen

**Affiliations:** 1 Department of Cardiology, First Affiliated Hospital of Xinjiang Medical University, Urumqi 830054, P.R. China; 2 Xinjiang Key Laboratory of Cardiovascular Disease Research, Urumqi 830054, P.R. China

**Keywords:** Cardiovascular disease, Risk factors, Disparities, Ethnicity, Epidemiology

## Abstract

**Background:**

Prevalence of cardiovascular disease (CVD) risk factors have been scarcely studied in Xinjiang, a multi-ethnic region.

**Methods:**

Multi-ethnic, cross-sectional cardiovascular risk survey study in Xinjiang, including individuals of Uygur (n = 4695), Han (n = 3717) and Kazakh (n = 3196) ethnicities, aged 35-74 years. Analyses involved 11,608 participants with complete data enrolled between October 2007 and March 2010.

**Results:**

There were differences in age-standardized prevalence of CVD risk factors between the three groups (all P < 0.001). Hypertension, obesity and smoking rates were higher among Kazakh (54.6%, 24.5%, and 35.8%, respectively). Dyslipidemia prevalence was higher among Uygur (54.3%), and diabetes prevalence was higher among Hans (7.1%). Age-standardized prevalence of adverse CVD risk profiles was different across different ethnicities. Compared with the Han participants, the Uygur and Kazakh had more CVD risk factors (P < 0.001). Compared with the Han participants, the adjusted odds ratios of 1, 2, and ≥3 risk factors profiles for Kazakh and Uygur participants were higher (all P < 0.001).

**Conclusions:**

The present study showed the pervasive burden of CVD risk factors in all participant groups in the Xinjiang region. Three major ethnic groups living in Xinjiang had striking differences in the prevalence of major CVD risk factors and adverse risk profiles. Ethnic-specific strategies should be developed to prevent CVD in different ethnic groups, as well as to develop strategies to prevent future development of adverse CVD risk factors at a younger age.

## Introduction

Cardiovascular diseases (CVD) account for about half of non-transmissible diseases deaths worldwide, namely 16.7 million 2002 [1]. In China, with the current shift toward an elderly population, CVD is an important and growing public health concern [2], accounting for nearly 40% of deaths in 1994 [3,4]. Furthermore, CVD incidence and mortality in China are projected to increase substantially during the next 20 years [3].

High numbers of CVD risk factors are common in many developed and developing countries, including China. These risk factors have emerged as important characteristics for predicting CVD morbidity and mortality [5]. The main strategy to prevent CVD is to control these risk factors, thus influencing the probability of developing CVD.

Recently, several studies have noted the striking differences in the prevalence of major CVD risk factors and adverse CVD risk profiles across ethnic groups in different parts of the world [6,7]. However, in China, these ethnic differences have been scarcely studied.

The present study aims to expand the literature on ethnic differences in CVD risk factors in China by describing the prevalence of five major and readily measurable CVD risk factors (high serum cholesterol or triglycerides and blood pressure levels, obesity, diabetes, cigarette smoking), and adverse CVD risk profiles (combinations of CVD risk factors; i.e., only one, two, or ≥ 3 risk factors) between three different ethnic groups (Han, Uygur and Kazakh) in the Xinjiang Uygur Autonomous Region (China), using data from the Cardiovascular Risk Survey (CRS).

## Methods

### Ethics statement

This study was approved by the Ethics Committee of the First Affiliated Hospital of Xinjiang Medical University and was conducted according to the standards of the Declaration of Helsinki. Written, informed consent was obtained from the participants.

### Sample design

The CRS study was a multi-ethnic, cross-sectional study designed to investigate the prevalence of risk factors for CVD, and to determine their contribution to atherosclerosis, coronary artery disease and cerebral infarction in the Chinese Han, Uygur, and Kazakh populations in Xinjiang (western China). Details about the sampling methods and design have been previously published [8-10]. Briefly, the CRS study used a 4-stage stratified sampling method to select a representative sample of the general population in Xinjiang. The research sites included seven cities (Urumqi, Kelamayi, Hetian, Zhaosu, Fukang, Tulufan, and Fuhai). The time period was from October 2007 to March 2010. The selections made from sampling units were based on geographic area, sex, and age groups using household registries. The 4-stage stratified sampling method was as follows: Stage one, according to population census data of Xinjiang in 2000, the area mentioned above were selected based on population, ethnicity, geography, economic and cultural development level respectively. Stage two, according to the ethnic aggregation status, one district or county was randomly selected from the Han, Uygur, Kazak population dominated area. Stage three, one community or town (village) was randomly selected from each district or county. Stages four, subjects aged above 35 years were randomly selected from each community or town (village) as research subjects. The staff conducted surveys in households and administered questionnaires. The questionnaires included the demographic, socioeconomic, dietary, and medical history of each participant. In total, the CRS included 14 618 participants (5757 Hans, 4767 Uyghur, and 4094 Kazakhs).

All analyses were restricted to survey participants without a history of myocardial infarction, stroke, or congestive heart failure (excluded n = 734), and missing some values (n = 993) were also excluded from the analyses, as well as 1283 participants being ≥ 75 years. Thus, these analyses were based on data from 11608 participants (3717 Han, 4695 Uygur and 3196 Kazakh people).

### Examination methods

Data collection was conducted in examination centers at the local hospital in the participants’ residential area. Before examination, participants were asked to fast, to refrain from smoking for 12 hours, and to avoid any vigorous physical activity. Height was measured to the nearest centimeter and body weight to the nearest 0.1 kg. Body mass index (BMI) was calculated as weight in kilograms divided by height in meters squared. After a 5-minute rest period, three seated blood pressure measurements were obtained using an automatic sphygmomanometer; the second and third readings were averaged. At the time of the in-person interview, a 5-mL blood sample was collected in an EDTA vacutainer tube. Plasma was separated within 30 min and stored at -80°C immediately. We measured fasting plasma glucose (FBG), triglycerides (TG), total cholesterol (TC), high-density lipoprotein cholesterol (HDL-C), and low-density lipoprotein cholesterol (LDL-C) levels using a Dimension AR/AVL Clinical Chemistry System (Newark, NJ, USA) in the Clinical Laboratory Department of the First Affiliated Hospital of Xinjiang Medical University.

### Cardiovascular risk factors

Major CVD risk factors were defined based on current national guidelines. Dyslipidemia was defined as TG ≥2.26 mmol/l, TC ≥ 6.22 mmol/l, LDL-C ≥ 4.14 mmol/l, HDL-C < 1.04 mmol/l, or if receiving a lipid-lowering drug [11]. Hypertension was defined as a systolic blood pressure (SBP) ≥ 140 mmHg, diastolic blood pressure (DBP) ≥ 90 mmHg, or if receiving an antihypertensive drug [12]. Obesity was defined as a BMI ≥30.0 [13]. Diabetes was defined as a fasting plasma glucose ≥6.99 mmol/l, or if using a diabetes drug [14]. Smoking was defined as currently smoking cigarettes.

### Statistical analyses

The statistical analysis was conducted using SPSS version 16.0 for Windows (SPSS Inc., Chicago, IL, USA). Age-standardization was performed by the direct method by using the 2000 Chinese population as the standard population.

The distribution of clinical characteristics among participants stratified by ethnic groups was analyzed using one-way ANOVA (with the least significant difference post hoc test) or chi-square tests. TG levels were log-transformed to normalize their distribution.

The age-standardized prevalence of each CVD risk factor (dyslipidemia, hypertension, diabetes, smoking, and obesity) was determined separately for men and women by ethnic group (Han, Uygur and Kazakh) and by age group (35-44, 45-54, 55-64, and 65-74 years).

The age-standardized prevalence of adverse CVD risk profiles (i.e., presence of 0, 1, 2 or ≥3 risk factors) were determined for the overall study population by ethnic groups (Han, Uygur and Kazakh), by age groups (35-44, 45-54, 55-64, and 65-74 years) and by gender, separately. The significance of the differences across subgroups was compared using the Wald X^2^test.

The adjusted odds ratios and 95% confidence intervals (95% CIs) of having one, two or ≥ 3 major CVD risk factors vs. no CVD risk factor were determined using multivariable logistic regression models that included ethnic group, age group and gender.

## Results

### Clinical characteristics

Table 1 presents the clinical characteristics of the participants. BMI, DBP, and TC were significantly higher in the Kazakh and Uygur groups compared with the Han group (all P < 0.001). FBG levels were significantly lower in the Kazakh and Uygur groups compared with the Han group (P < 0.001). SBP and HDL-C levels were highest in the Kazakh (P < 0.05), which had no difference in the Han and Uygur groups. TG levels were log-transformed to normalize their distribution, and significantly higher for Uygur and Han than Kazakh (P < 0.001), but there was no difference between Uygur and Han. And no difference was observed in LDL-C levels between the three groups.

**Table 1:** Characteristics of all participants and by ethnic groups in Xinjiang

	**Total**	**Han**	**Uygur**	**Kazakh**
	**N=11,608**	**N=3,717**	**N=4,695**	**N=3,196**
Age (y)	51.1±10.5	51.3±10.3	51.9±11.0	49.7±10.0
Gender (female, %)	6,337 (54.6)	2,179 (58.6)	2,493 (53.1)	1,665 (52.1)
BMI (kg/m^2^)*	25.9±4.2	25.1±3.5	26.0±4.4	26.8±4.8
SBP (mmHg)&	134.9±22.2	132.2±21.0	132.4±19.7	141.9±25.2
DBP (mmHg)*	85.2±16.8	81.0±14.7	85.4±15.5	89.8±19.6
FBG (mmol/L)#	5.15±1.69	5.32±1.80	4.94±1.65	5.16±1.55
TG (mmol/L)$	1.27±0.82	1.39±0.84	1.39±0.78	1.01±0.37
TC (mmol/L)*	4.64±1.12	4.39±1.09	4.71±1.08	4.82±1.17
HDL-C (mmol/L)&	1.26±0.46	1.26±0.48	1.25±0.46	1.29±0.43
LDL-C (mmol/L)**	2.88±0.92	2.86±0.92	2.87±0.92	2.90±0.93

*SBP* systolic blood pressure, *DBP* diastolic blood pressure, *BMI* body mass index, *TC* serum total cholesterol, *TG* triglyceride, *LDL-C* low-density lipoprotein cholesterol, *HDL-C* high-density lipoprotein cholesterol, *FBG* fasting blood glucose.

The differences between ethnic groups were performed by F-test or by χ^2^test.

*Significantly higher for Kazakh and Uygur than Han, there were differences between the three groups. (All P < 0.001).

&Significantly higher for Kazakh than Uygur and Han (P < 0.05), but there was no difference between Uygur and Han.

#Significantly higher for Han than Uygur and Kazakh, there were differences between the three groups. (All P < 0.001).

$Levels were log-transformed to normalize their distribution, and significantly higher for Uygur and Han than Kazakh (P < 0.001), but there was no difference between Uygur and Han.

**No difference between the three groups (P > 0.05).

### Prevalence of CVD risk factors in Xinjiang

In the Xinjiang population aged 35-74 years, the age-standardized prevalence of hypertension, diabetes, obesity, dyslipidemia and smoking were 44.2%, 5.5%, 15.6%, 51.7% and 28.6%, respectively. In Han participants, prevalence of these diseases was 36%, 7.1%, 8.1%, 53.3% and 19.3%, respectively. In Uygur participants, prevalence of these diseases was 43.7%, 5.4%, 17.6%, 54.3% and 31.1%. Finally, in Kazakh participants, prevalence of these diseases was 54.6%, 3.4%, 24.5%, 45.9% and 35.8% (Table 2).

**Table 2:** Age- and gender-standardized prevalence of CVD risk factors in Xinjiang by ethnic groups

**Ethnic groups (n)**	**Hypertension (%)**	**Diabetes (%)**	**Obesity (%)**	**Dyslipidemia (%)**	**Smoking (%)**
Total					
Overall(11608)	44.2	5.5	15.6	51.7	28.6
35-44y(3999)	28.1	3.1	12.7	48.0	30.6
45-54y(3339)	43.8	3.2	17.3	52.9	29.7
55-64y(2559)	56.2	6.6	18.6	54.4	26.1
65-74y(1711)	65.0	8.4	14.7	53.5	25.6
Men(5271)	46.5	6.6	15.3	54.5	56.3
Women(6337)	42.3	4.6	15.9	49.3	5.6
Han					
Overall(3717)	36.0	7.1	8.1	53.3	19.3
35-44y(1158)	19.1	3.8	5.8	49.3	18.1
45-54y(1109)	37.5	8.4	6.5	53.1	20.1
55-64y(952)	46.0	8.1	11.7	58.4	20.1
65-74y(498)	52.6	10.4	10.4	53.6	19.1
Men(1583)	35.1	8.4	8.9	54.4	45.4
Women(2179)	36.6	6.0	7.3	52.6	1.0
Uygur					
Overall(4695)	43.7	5.4	17.6	54.3	31.1
35-44y(1643)	29.2	2.8	16.2	51.6	32.9
45-54y(1228)	37.7	5.5	20.0	57.1	35.2
55-64y(948)	55.8	7.6	17.5	54.5	26.5
65-74y(876)	66.1	7.2	15.3	55.1	26.9
Men(2202)	46.5	6.0	14.8	58.8	63.6
Women(2493)	41.2	5.0	19.6	50.3	2.4
Kazakh					
Overall(3196)	54.6	3.4	24.5	45.9	35.8
35-44y(1198)	35.1	2.5	18.8	42.0	39.6
45-54y(1002)	58.2	4.2	27.3	47.6	33.7
55-64y(659)	71.3	3.2	30.2	48.6	34.1
65-74y(337)	80.7	5.0	24.9	49.3	31.8
Men(1531)	58.1	4.8	25.1	48.3	56.6
Women(1665)	51.4	2.2	23.8	43.6	16.6

Hypertension was defined as SBP ≥ 140 mmHg and/or DBP ≥ 90 mmHg and/or current antihypertensive drug use. Diabetes was defined as fasting plasma glucose ≥ 6.99 mmol/L and/or current diabetes drug use. Obesity was defined as BMI ≥ 30 kg/m^2^. Dyslipidemia was defined as TG ≥ 2.26 mmol/L, TC ≥ 6.22 mmol/L, HDL-C < 1.04 mmol/L, LDL-C ≥ 4.14 mmol/L, and/or current lipid-lowering drug use.

The age-standardized prevalence of hypertension, diabetes, dyslipidemia and smoking was higher in men than in women (all P < 0.001). However, in Uygur participants, the prevalence of obesity was higher in women than in men (P < 0.001) (Table 2).

Hypertension and diabetes prevalence increased with age, while smoking decreased with age among all participants (all P < 0.001). Among Han and Uygur participants, hypertension prevalence increased for the entire age range (all P < 0.001). Among Kazakh participants, hypertension and dyslipidemia prevalence increased with age (all P <0.001) (Table 2).

The age-standardized prevalence of hypertension, obesity and smoking was significantly higher in Uygur and Kazakh than compared with Han participants (all P < 0.001). Diabetes prevalence was higher in Han participants, and lower in Kazakh participants. Dyslipidemia prevalence was higher in Uygur participants, and lower in Kazakh participants (Table 2).

### Prevalence of adverse CVD risk profiles in Xinjiang

**Figure 1:**
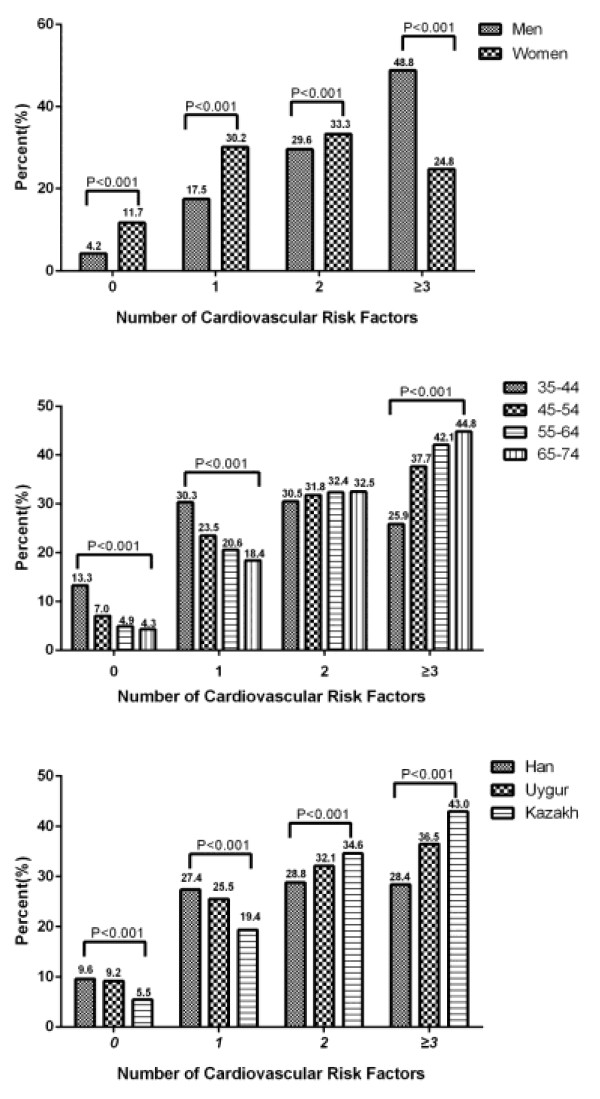
Age-standardized prevalence of adverse CVD risk profiles among Xinjiang participants stratified by gender groups (top), age groups (middle), and ethnic groups (bottom).

Overall, 17.5% of men had only one major risk factor, while 29.5% and 48.8% had two or three or more risk factors, respectively. Among women, 30.2% had only one risk factor, while 33.3% and 24.8% had two or three or more risk factors, respectively (Figure 1).

Overall, 13.3% of participants aged 35-44 years did not have any of the investigated risk factors, while 25.9% had three or more risk factors. By contrast, 4.3% of participants aged 65-74 did not have any of the investigated risk factors, while 44.8% had three or more risk factors. The number of risk factors increased with age (Figure 1).

Overall, 9.6%, 27.4%, 28.8% and 28.4% of Han, 9.2%, 25.5%, 32.1% and 36.5% of Uygur, and 5.5%, 19.4%, 34.6% and 43.0% of Kazakh participants had none, one, two or three or more risk factors, respectively. In summary, compared with the Han participants, the Uygur and Kazakh had more risk factors (Figure 1).

Compared with the Han participants, the adjusted odds ratio of having one, two, or three or more CVD risk factors for the Uygur participants were 1.16, 1.29 and 1.55; and were 1.72, 2.75 and 4.03, respectively, for the Kazakh participants. The adjusted odds ratio of having CVD risk factors vs. none increased progressively with increasing age. The adjusted odds ratios for having one, two, or three or more CVD risk factors for subjects aged 65-74 years compared with patients aged 35-44 were 2.23, 4.27 and 4.82, respectively. In addition, males were more likely to have one, two, or three or more CVD risk factors compared with females(all P < 0.001)(Table 3).

**Table 3:** Adjusted* odds ratios (95% CI) of having one, two or three or more vs. none CVD risk factors according to gender, age, and ethnic groups

	**1 risk factor**	**2 risk factors**	**≥3 risk factors**
Ethnic groups			
Han	1.00 (ref)	1.00 (ref)	1.00 (ref)
Uygur	1.16 (1.03-1.29)	1.29 (1.14-1.46)	1.55 (1.32-1.83)
Kazakh	1.72 (1.50-1.97)	2.75 (2.37-3.18)	4.03 (3.37-4.82)
Age groups			
35-44y	1.00 (ref)	1.00 (ref)	1.00 (ref)
45-54y	1.30 (1.15-1.47)	2.00 (1.75-2.29)	2.26 (1.91-2.69)
55-64y	1.90 (1.65-2.19)	3.50 (3.00-4.08)	3.96 (3.28-4.79)
65-74y	2.23 (1.87-2.65)	4.27 (3.56-5.13)	4.82 (3.87-6.00)
Male gender	1.96 (1.76-2.18)	3.54 (3.15-3.97)	6.55 (5.68-7.56)

*All variables (age, gender and ethnic groups) were included in the same models; thus the odds ratios for age groups were adjusted for gender and ethnic group; the odds ratios for gender were adjusted for age groups and ethnic group; and the odds ratios for ethnic grouping were adjusted for age groups and gender.

## Discussion

The present study provides several insights about CVD risk factors among Chinese Han, Uygur, and Kazakh participants in Xinjiang. The results of the present study indicated that in Han participants, the age-standardized prevalence of hypertension, diabetes, obesity, dyslipidemia and smoking was 36%, 7.1%, 8.1%, 53.3% and 19.3%, respectively. In Uygur participants, prevalence of these diseases was 43.7%, 5.4%, 17.6%, 54.3% and 31.1%. In Kazakh participants, prevalence of these diseases was 54.6%, 3.4%, 24.5%, 45.9% and 35.8%. Furthermore, 9.6%, 27.4%, 28.8% and 28.4% of Han, 9.2%, 25.5%, 32.1% and 36.5% of Uygur, and 5.5%, 19.4%, 34.6% and 43.0% of Kazakh participants had none, one, two or three or more risk factors, respectively. Finally, compared with the Han participants, the adjusted odds ratios of 1, 2, and ≥3 risk factors profiles for Kazakh and Uygur participants were higher.

Several studies have noted the striking differences across ethnic groups in the prevalence of CVD risk factors in the world [15-17]. In this study, we observed that the prevalence of individual major CVD risk factors varied markedly across these three different ethnic groups. Hypertension, obesity and smoking rates were higher among Kazakh. Dyslipidemia prevalence was higher among Uygur, and diabetes prevalence was higher among Hans. Moreover, age-standardized prevalence of adverse CVD risk profiles was different across different ethnic groups. Compared with the Han participants, Uygur and Kazakh participants were more likely to have multiple CVD risk factors, reflecting the fact that all of the investigated CVD risk factors except diabetes were more frequent in Uygur or Kazakh participants.

Ethnicity is a social construct, a concept that intertwines biological, sociocultural, psychological and behavioral components. All ethnic groups can share a range of phenotypic characteristics due to the shared ancestry; the term ethnicity is typically used to highlight cultural and social characteristics such as language, ancestry, religious traditions, dietary preferences and history. The study of Latinos showed [18] that age-standardized prevalence of CVD risk factors varied by Hispanic/Latino background, prevalence of adverse CVD risk profiles was higher among participants with Puerto Rican background, which had lower socioeconomic status, and higher levels of acculturation. Another study in India [19] found marked differences in conventional risk factors between the Meitei and the Aggarwal, since the genetic and cultural backgrounds are different for both groups.

The mechanisms underlying the striking differences across ethnic groups in the prevalence of hypertension, diabetes, dyslipidemia, obesity and smoking are not clear. In this study, it is believed that different environmental exposures among Chinese Han, Uygur and Kazakh ethnic groups may play an important role. Beside the Han participants, the inhabited area of Chinese Uygur and Kazakh participants is relatively isolated and fixed. Most Kazakh people live as animal raisers and reside in the villages and forests north of Xinjiang, which are cold and semiarid, while most Uygur people live as farmers in the plains south of Xinjiang, which are hot and arid. Moreover, Chinese Uygur and Kazakh share similar dietary habits, characterized by drinking strong wine, eating more animal fat, a higher salt intake and consuming less grain, fresh vegetables, beans, bean products, and unsaturated fatty acids [20]. In addition to different environmental exposures among Chinese Han, Uygur and Kazakh ethnic groups, differences in genetic backgrounds and gene-environment interactions could also be important factors underlying the different prevalence of hypertension [21-24] and diabetes [25]. Further studies between these adverse CVD risk profiles and ethnic-specific genetic susceptibility are needed to clarify this observation.

Several studies in the United States have investigated the impact of adverse risk profiles on CVD incidence, mortality, and quality of life [26-30]. In these studies, CVD incidence and all-cause mortality increased progressively and substantially in the presence of more risk factors. For example, data from the First NHANES Epidemiologic Follow-up Study showed that the age-,race-, sex-, and education-adjusted relative risks of CVD during the 21-year follow-up in adults with one, two, three, four or five risk factors were 1.6,2.2, 3.1, and 5.0, respectively, vs. participants without any risk factor [30]. In longitudinal studies, the presence of CVD risk factors at baseline has been associated with a diminished quality of life [26]. These programs to enhance efforts aimed at prevention, detection, and treatment of dyslipidemia, hypertension, diabetes, smoking, and obesity may greatly reduce the future burden of CVD.

The striking differences across ethnic groups with regard to the prevalence of CVD risk factors and adverse CVD risk profiles emphasize the need for the development of ethnic-specific and cost-effective CVD prevention programs and health services to reduce the prevalence of these risk factors, as well as CVD morbidity and mortality in the Chinese Han, Uygur and Kazakh participants in Xinjiang. In addition, future public health interventions need to take into account the special needs of people living in Xinjiang. Effective interventions such as smoking cessation, improved diet (reduction of salt and fat), and increased physical activity can safely and effectively lower the risk of CVD [31]. A multidisciplinary and targeted approach aiming at prevention, detection and treatment of hypertension, dyslipidemia, diabetes, obesity and smoking could substantially reduce the prevalence of adverse CVD risk profiles, as well as CVD morbidity and mortality.

Participants with a history of myocardial infarction, stroke, or congestive heart failure were excluded from our analyses in order to focus on the burden of CVD risk factors among patients without CVD. Nonetheless, results were markedly consistent when participants with existing CVD complications were included in the analyses. We chose not to include physical inactivity as a CVD risk factor in the current analyses because it is causally involved in the development of all CVD risk factors studied, except cigarette smoking. Inclusion of physical inactivity as a risk factor would have artificially inflated the prevalence of CVD risk factor profiles. The findings of the present study are limited to self-reported information. However, the data were age-standardized to the Chinese population in 2000 to allow for comparisons with observations from national surveys, and protocols used were similar to those of other epidemiological studies.

In conclusion, the present study demonstrated the pervasive burden of CVD risk factors in all participant groups in Xinjiang and identified specific ethnic groups at particularly high risk of CVD. These data may increase the need to implement interventions to lower the burden of CVD risk factors among Xinjiang people overall. Ethnic-specific strategies should be developed to prevent CVD in different ethnic groups, as well as strategies to prevent future development of adverse CVD risk factors starting at the youngest ages.

## Competing interests

The authors declare that they have no competing interests.

## Authors’ contributions

Conceived and designed the experiments: JT, YT-M, YX. Physical, biochemical measurements and data collecting: JT, YT-M, YX, YN-Y, XM, ZY-F, XM-L, XX, YC, ZX-Y, BD-C. Analyzed the data: JT, YX, FL. Wrote the paper: TJ, YT-M, YX. All authors read and approved the final manuscript.
